# Effects of haloperidol inhalation on MK-801- and memantine-induced locomotion in mice

**DOI:** 10.1080/19932820.2020.1808361

**Published:** 2020-08-18

**Authors:** Hiroshi Ueno, Shunsuke Suemitsu, Shinji Murakami, Naoya Kitamura, Kenta Wani, Yu Takahashi, Yosuke Matsumoto, Motoi Okamoto, Takeshi Ishihara

**Affiliations:** a Department of Medical Technology, Kawasaki University of Medical Welfare, Okayama, Japan; b Department of Psychiatry, Kawasaki Medical School, Okayama, Japan; c Department of Neuropsychiatry, Graduate School of Medicine, Dentistry and Pharmaceutical Sciences, Okayama University, Okayama, Japan; d Department of Medical Technology, Graduate School of Health Sciences, Okayama University, Okayama, Japan

**Keywords:** Inhalation, dizocilpine, haloperidol, memantine, mouse, schizophrenia

## Abstract

The administration of therapeutic agents is difficult in many patients, such as patients with post-operative delirium or dementia or patients with schizophrenia, who are upset in an emergency room. Therefore, the development of a new method for administering therapeutic agents to the central nervous system is desired. In this study, we investigated if inhalation was an effective route of administration for haloperidol, a commonly used, strong antipsychotic. Dizocilpine, also known as MK-801, is a noncompetitive antagonist of the N-methyl-D-aspartate receptor. MK-801 or memantine-induced motor hyperactivity was evaluated in mice following either intraperitoneal injection or inhalation of haloperidol or the histamine neuroactivator betahistine. Pretreatment with haloperidol inhalation inhibited the MK-801-induced or memantine-induced increase in locomotor activity. This effect was similar to that of the intraperitoneal administration of haloperidol. However, pretreatment with inhaled betahistine or the intraperitoneal administration of betahistine did not suppress the MK-801-induced or memantine-induced increase in locomotor activity. Thus, haloperidol when inhaled acts on the central nervous system of mice and suppresses the MK-801-induced increase in mouse locomotor activity. Our findings suggest that inhalation may be a novel method for administering haloperidol.

**Abbreviations:**

ANOVA: analysis of variance

## 1. Background

Despite many years of research, most neuropsychiatric disorders have unmet therapeutic needs. The complex pathophysiology of brain injury and the difficulty of accessing the brain using small and large drug molecules are major obstacles in the research and development of new treatments [1]; in addition, the risk, complexity, and cost of clinical trials required for their regulatory approval present significant hurdles as well. Therefore, the discovery of new and effective therapeutic agents for the central nervous system (CNS) is of critical importance [2]. The management of patients with schizophrenia in an emergency room requires medications that are rapidly effective, and oral or intravenous administration is impractical. An intramuscular injection of ziprasidone, olanzapine, and aripiprazole provides more rapid mental stabilization but requires 1–3 hours to achieve maximal concentration. Intramuscular administration can be dangerous for patients who are agitated and also for health-care professionals tending to them in the emergency room. Therefore, new drug delivery methods for antipsychotic drugs to treat acute excitement are desired. The administration of drugs by inhalation, as presented in this study, may be useful. Furthermore, the administration of therapeutic agents by inhalation may be useful even when it is difficult for a patient to take drugs because of conditions such as post-operative delirium or dementia. The development of therapeutics to the CNS involves overcoming many factors, such as the blood-brain barrier (BBB), degradation in the gastrointestinal tract and/or metabolism in the liver, and gastrointestinal tract or systemic adverse events [3]. Injection is one of the alternative routes for administering drugs into the blood; however, it is associated with drawbacks such as pain, scar tissue formation with frequent dosing, and needle phobia in children. A novel approach to circumventing this problem is to use a non-invasive method that can bypass the blood-brain barrier. Nasal delivery is one such method, which has the potential to avoid systemic circulation and deliver drugs directly to the brain [5,8].

The neurofilaments of the olfactory nerve or axons extending directly from the olfactory bulb in the marginal area of the brain to the upper back of the nose penetrate the mucosal lining and make direct contact with the outside world. This unique and important anatomical arrangement may provide a potential pathway for direct drug access to the CNS [7,10,^9^]. In fact, some drugs are already administered intranasally, such as sumatriptan for migraine headaches and desmopressin for the treatment of diabetes mellitus in response to lactation [10,13]. Current nasal administration protocols require that a liquid containing the drug is inhaled using a dedicated device [12,15]. In this study, we investigated whether delivering a drug to the brain is possible by inhalation without using a dedicated device (e.g., inhalation using aromatherapy). In this study, mice inhaled the CNS depressants haloperidol and betahistine using a nebulizer. The mechanism of action of the nebulizer is the same mechanism which is used in aromatherapy to convert essential oils into fine air particles for inhalation.

Schizophrenia is a severe and chronic mental disorder that affects 0.5–1% of the general population [14,17]. Based on the *N*-methyl-d-aspartate (NMDA) hypofunction hypothesis of schizophrenia [16–18], the NMDA receptor antagonist MK-801 is widely used in rodents to induce schizophrenia-like behavioral abnormalities, including positive and negative symptoms and cognitive impairment [19–25]. The blockade of glutamatergic transmission by MK-801 induces robust and dose-dependent increases in locomotor activity in mice [26]. If a novel compound can alleviate MK-801-induced behavioral abnormalities, then it is widely used as part of a series of trials to evaluate its preclinical utility as a potential antipsychotic agent [27–29]. In this study, changes in mouse locomotor activity induced by MK-801 administration were investigated.

Memantine (1-amino-3,5-dimethyladamantane) is an amantadine derivative that functions as a voltage-dependent non-competitive antagonist of the glutamatergic NMDA receptor [30]. Memantine blocks serotonin (5-HT3) receptors [31,34] and α7 nicotinic acetylcholine receptors [33], but activates dopamine D2 receptors [34]. Memantine-induced hyperactivity increases significantly in a dose-dependent manner [35]. In this study, changes in the locomotor activity of mice after memantine administration were also investigated.

First-generation antipsychotics such as haloperidol and chlorpromazine are used to treat schizophrenia, and have shown efficacy in alleviating the psychotic symptoms of schizophrenia [36]. Haloperidol produces selective effects on the CNS by the competitive blockade of postsynaptic dopamine (D2) receptors in the mesolimbic dopaminergic system [37]. This drug can especially act on the D2 receptors in the nigrostriatal dopaminergic pathway [38]. In adult rats, haloperidol also causes catalepsy and motor depression with acute administration; this effect can be used as behavioral confirmation of its successful delivery to the brain [39].

Betahistine (2- [2- (methylamino) ethyl] pyridine) is a drug used to treat vertigo associated with Ménière disease [40]. This drug is a potent H3 receptor antagonist [41]. It has been reported that brain histamine levels increase with memantine-induced hyperactivity [42]. In mice, betahistine attenuates the increase in locomotor activity induced by memantine [35].

In this study, the effect of inhaled betahistine on the suppression of hyperactivity in mice was also examined. The purpose of this study was to clarify whether the inhalation of CNS depressants such as haloperidol and betahistine suppresses MK-801-induced and memantine-induced increases in locomotor activity.

## 2. Methods

### 2.1. Animals

Fifteen-week-old male mice (C57BL/6) were purchased from Charles River Laboratories (Kanagawa, Japan) and housed in cages with food and water provided *ad libitum* under a 12-h light/dark cycle at 23–26°C. Every effort was made to minimize the number of mice used and their suffering. These experiments complied with the U.S. National Institutes of Health (NIH; Bethesda, MD, USA) Guide for the Care and Use of Laboratory Animals (NIH Publication No. 80–23, revised in 1996). The experiments were approved by the Committee for Animal Experiments at Kawasaki Medical School Advanced Research Center (Kurashiki, Japan). Each animal was subjected to experimental manipulations only once (7–10 mice per group). The animals were sacrificed by CO_2_ inhalation.

### 2.2. Reagents

(+)-MK-801 (130–17,381), Memantine (131–18,313), and Haloperidol (084–04261) were purchased from Fujifilm Wako Pure Chemical Corporation (Osaka, Japan) and Betahistine (B1424) was purchased from Tokyo Chemical Industry Co., Ltd. (Tokyo, Japan). All reagents were diluted in saline and administered intraperitoneally. (+)-MK-801 was diluted to a concentration of 0.1 mg/mL and administered at a dose of 0.2 mg/kg. This dose was selected based on previous studies demonstrating a schizophrenia-related deficient effect of MK-801 at 0.2 mg/kg in mice [43–45]. Memantine was diluted at a concentration of 10 mg/mL and administered at a dose of 20 mg/kg. This dose was selected based on previous studies demonstrating a hyperlocomotive effect of memantine at 20 mg/kg in mice [35]. Haloperidol was dissolved in saline with a minimal amount of acetic acid [46] and administered at a dose of 2 mg/kg, whereas, betahistine was diluted at a concentration of 10 mg/mL and administered at a dose of 10 mg/kg [35]. The same amount of acetic acid was added to the vehicle for control animals.

### 2.3. Inhalation of the reagents

**Figure 1.**
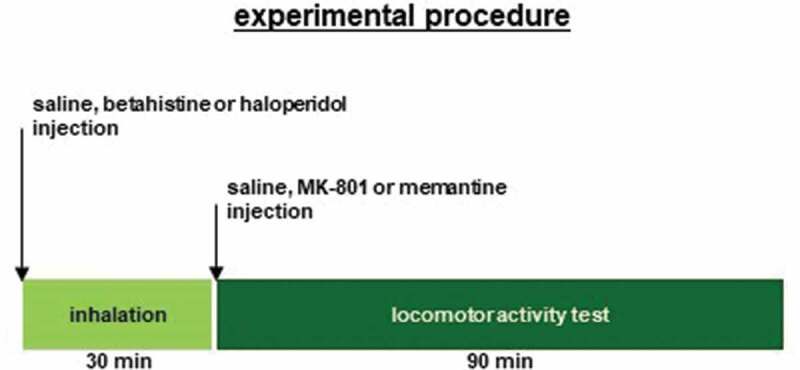
Experimental procedure Mice were treated with saline, haloperidol, or betahistine 30 minutes before the i.p. injection of saline, MK-801, or memantine. After injection, all mice were acclimated in a single housing environment for 90 minutes for locomotor activity measurements.

The inhalation apparatus was the same as the apparatus used in a previous study [47]. Mice were exposed to each reagent by means of a mesh nebulizer (NEB-01; Custom Corporation, Tokyo, Japan). The reagent was inhaled in a sealed container. The nebulizer was placed on a stainless-steel wire bar lid on a new breeding cage (235 mm × 325 mm × 170 mm) surrounded by two larger cages (292 mm × 440 mm × 200 mm). The mice were unable to lick the reagents. Approximately 5 minutes after placing the nebulizer, the mice were placed in the internal cage. The mice inhaled 1 mg/mL of haloperidol, 10 mg/mL of betahistine, or saline 30 minutes before the behavioral test (Figure 1).

### 2.4. Locomotor activity test

All behavioral experiments were conducted during the light phase (9:00–16:00). We tested mice in different groups in random order. After testing, the apparatus was cleaned with 70% ethanol and water with super-oxidized hypochlorous acid to prevent any bias due to olfactory cues. For the measurement of locomotor activity, the mice were acclimated to a single housing environment. Locomotor activity data were measured using a photobeam activity system (ACTIMO-100; BRC Co., Nagoya, Aichi, Japan), and activity counts were recorded at 10-min intervals.

### 2.5. Effects of administering MK-801 and memantine on mouse locomotor activity

To measure locomotor activity, the mice were first acclimated to a single housing environment for 120 minutes, during which locomotor activity was measured for 30 minutes to allow the animals to get used to the experimental equipment. Thereafter, the mice were randomly divided into three groups (n = 8 per group), and treated i.p. with 0.2 mg/kg of MK-801, 20 mg/kg of memantine, or saline. Locomotor activity was then measured 120 minutes after administration.

### 2.6. Effects of haloperidol on MK-801-induced hyperlocomotion

The mice were randomly divided into four groups: (1) saline inhalation and i.p. saline injection (n = 6), (2) saline inhalation and i.p. MK-801 injection (n = 8), (3) haloperidol inhalation and i.p. MK-801 injection (n = 8), and (4) i.p. haloperidol injection and i.p. MK-801 injection (n = 8). The mice were treated with inhaled saline or haloperidol or i.p. with haloperidol 30 minutes before the injection of saline or MK-801. After injection, all mice were acclimated to a single housing environment for 90 minutes. The measurement time was 90 minutes. This test did not allow the mice time to get used to the experimental setup.

### 2.7. Effects of haloperidol on memantine-induced hyperlocomotion

The mice were randomly divided into four groups: (1) saline inhalation and i.p. saline injection (n = 6), (2) saline inhalation and i.p. memantine injection (n = 8), (3) haloperidol inhalation and i.p. memantine injection (n = 8), and (4) i.p. haloperidol injection and i.p. memantine injection (n = 8). Mice were treated with inhaled saline or haloperidol or treated i.p. with haloperidol 30 minutes before the injection of saline or memantine. After injection, all mice were acclimated to a single housing environment for 90 minutes. The measurement time was 90 minutes. This test did not allow the mice time to get used to the experimental setup.

### 2.8. Effects of betahistine on MK-801-induced hyperlocomotion

The mice were randomly divided into four groups: (1) saline inhalation and i.p. saline injection (n = 8), (2) saline inhalation and i.p. MK-801 injection (n = 8), (3) betahistine inhalation and i.p. MK-801 injection (n = 8), and (4) i.p. betahistine injection and i.p. MK-801 injection (n = 8). Mice were treated with inhaled saline or betahistine or treated i.p. with betahistine 30 minutes before injection with saline or MK-801. After injection, all mice were acclimated to a single housing environment for 90 minutes. The measurement time was 90 minutes. This test did not allow the mice time to get used to the experimental setup.

### 2.9. Effects of betahistine on memantine-induced hyperlocomotion

The mice were randomly divided into the four groups: (1) saline inhalation and i.p. saline injection (n = 8), (2) saline inhalation and i.p. memantine injection (n = 8), (3) betahistine inhalation and i.p. memantine injection (n = 8), and (4) i.p. betahistine injection and i.p. memantine injection (n = 8). Mice were treated with inhaled saline or betahistine or treated i.p. with betahistine 30 minutes before injection with saline or memantine. After injection, all mice were acclimated to a single housing environment for 90 minutes. The measurement time was 90 minutes. This test did not allow the mice time to get used to the experimental setup.

### 2.10. Statistical analyses of behavioral test results

Data were analyzed using a two-way analysis of variance (ANOVA) followed by Tukey’s test or with a two-way repeated measures ANOVA followed by Fisher’s least significant difference test. Statistical analyses were performed using SPSS software (IBM, Armonk, NY, USA). Differences with *p* < 0.05 were deemed statistically significant. Data are presented as the mean ± the standard error of the mean or in box plots.

## 3. Results

### 3.1. Locomotor activity in mice after MK-801 or memantine administration

**Figure 2.**
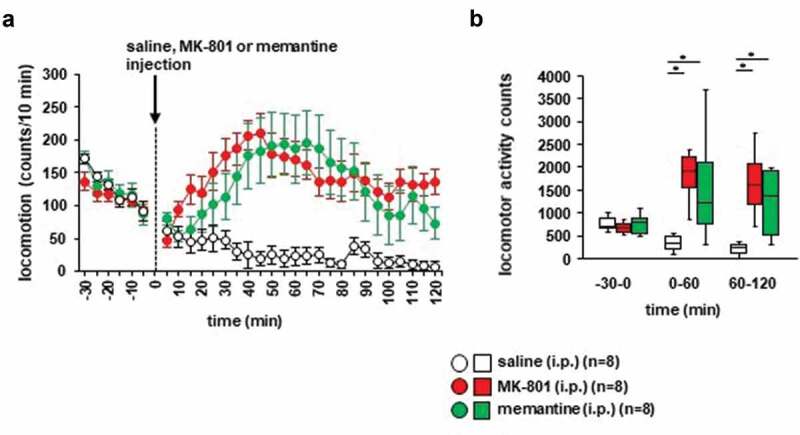
The effect of MK-801 and memantine on mouse locomotion (a) Spontaneous locomotor activity for each 10-minute period. After 30 minutes, the mice were injected with MK-801, memantine, or saline. Locomotor activity was then assessed for 120 minutes. (b) Total beam breaks for 30 minutes before the administration of MK-801, memantine, or saline, and for each 60-minute period after administration. Data are presented as **A** the mean ± SEM or **B** box plots.

First, we examined whether the locomotor activity of the mice was affected by i.p. MK-801 and memantine administration compared to i.p. saline administration. As shown in Figure 2, the injection of MK-801 or saline caused a robust increase in locomotor activity, which lasted for 120 minutes (Figure 2(a): *F*_29,434_ = 6.074, *p* < 0.001; saline vs. MK-801, *p* = 0.008; saline vs. memantine, *p* = 0.017; MK-801 vs. memantine, *p* = 0.939; Figure 2(b), for −30 minutes to 0 minutes: *F*_2,23_ = 0.518, *p* = 0.603; saline vs. MK-801, *p* = 0.652; saline vs. memantine, *p* = 1.0; MK-801 vs. memantine, *p* = 0.664; for 0–60 minutes: *F*_2,23_ = 7.615, *p* = 0.003; saline vs. MK-801, *p* = 0.004; saline vs. memantine, *p* = 0.019; MK-801 vs. memantine, *p* = 0.765; for 60–120 minutes: *F*_2,23_ = 6.764, *p* = 0.005; saline vs. MK-801, *p* = 0.009; saline vs. memantine, *p* = 0.016; and MK-801 vs. memantine, *p* = 0.972). For 120 minutes post-injection of saline, locomotor activity counts were significantly higher in mice injected with MK-801 and memantine than in mice injected with saline (Figure 2(b)).

* Indicates a significant difference between groups (*p *< 0.05). The *p* values were calculated using (a two-way repeated measures analysis of variance and **B** two-way analysis of variance. (**A–B**) control: n = 8, MK-801: n = 8, memantine: n = 8.

### 3.2. Effects of haloperidol inhalation on MK-801–induced locomotor activity in mice

**Figure 3.**
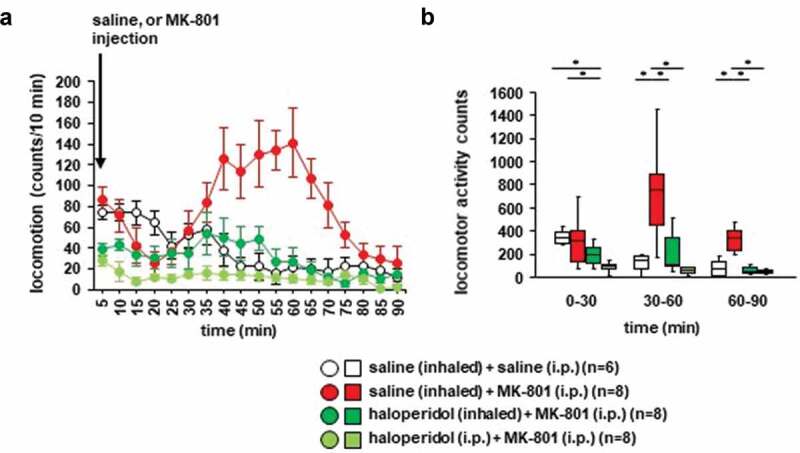
Effect of haloperidol inhalation on MK-801–induced locomotion Mice were pretreated with inhaled saline or haloperidol or i.p. with haloperidol 30 minutes before the MK-801 injection. a) After i.p. MK-801 administration, spontaneous locomotor activity of the mice was measured for each 5-minute period for 90 minutes in the locomotor activity test. b) The graphs show the total beam breaks for each 30-minute period after administration.

Whether haloperidol inhalation affected MK-801-induced locomotor activity was examined. Twenty minutes after the MK-801 injection, locomotor activity gradually increased (Figure 3(a): *F*_17,476_ = 5.093, *p* < 0.001; saline vs. MK-801, *p* = 0.014; saline vs. haloperidol (inhaled), *p* = 0.933; saline vs. haloperidol (i.p.), *p* = 0.179; MK-801 vs. haloperidol (inhaled), *p* = 0.003; MK-801 vs. haloperidol (i.p.), *p* < 0.001; haloperidol (inhaled) vs. haloperidol (i.p.), *p* = 0.449). In contrast, locomotor activity gradually decreased after the injection of saline. Haloperidol pretreatment (i.e., injection of haloperidol or inhalation of haloperidol) prevented the increase of locomotor activity induced by MK-801 (Figure 3(a,b), for 0–30 minutes: *F*_3,31_ = 6.101, *p* = 0.002; saline vs. MK-801, *p* = 0.825; saline vs. haloperidol (inhaled), *p* = 0.126; saline vs. haloperidol (i.p.), *p* = 0.002; MK-801 vs. haloperidol (inhaled), *p* = 0.494; MK-801 vs. haloperidol (i.p.), *p* = 0.020; haloperidol (inhaled) vs. haloperidol (i.p.), *p* = 0.341; for 30–60 minutes: *F*_3,31_ = 9.669, *p* < 0.001; saline vs. MK-801, *p* = 0.001; saline vs. haloperidol (inhaled), *p* = 0.946; saline vs. haloperidol (i.p.), *p* = 0.877; MK-801 vs. haloperidol (inhaled), *p* = 0.005; MK-801 vs. haloperidol (i.p.), *p* < 0.001; haloperidol (inhaled) vs. haloperidol (i.p.), *p* = 0.574; for 60–90 minutes: *F*_3,31_ = 13.599, *p* < 0.001; saline vs. MK-801, *p* = 0.001; saline vs. haloperidol (inhaled), *p* = 0.900; saline vs. haloperidol (i.p.), *p* = 0.554; MK-801 vs. haloperidol (inhaled), *p* < 0.001; MK-801 vs. haloperidol (i.p.), *p* < 0.001; haloperidol (inhaled) vs. haloperidol (i.p.), *p* = 0.919). No significant difference was observed in the locomotor activity counts between mice exposed to haloperidol injection and mice exposed to haloperidol inhalation (Figure 3(a,b)). Administration of haloperidol 30 minutes before the injection of MK-801 did not increase locomotor activity. For 30 minutes after administration, the total locomotor activity count was reduced in mice injected with haloperidol compared with mice injected with saline (Figure 3(b)).

* Indicates a significant difference between groups (*p* < 0.05). The *p* values were calculated using **A** two-way repeated measures analysis of variance and **B** two-way analysis of variance.(**A–B**) control: n = 6, MK-801: n = 8, haloperidol (inhaled): n = 8, haloperidol (i.p.): n = 8.

### 3.3. Effects of haloperidol inhalation on memantine-induced locomotor activity in mice

**Figure 4.**
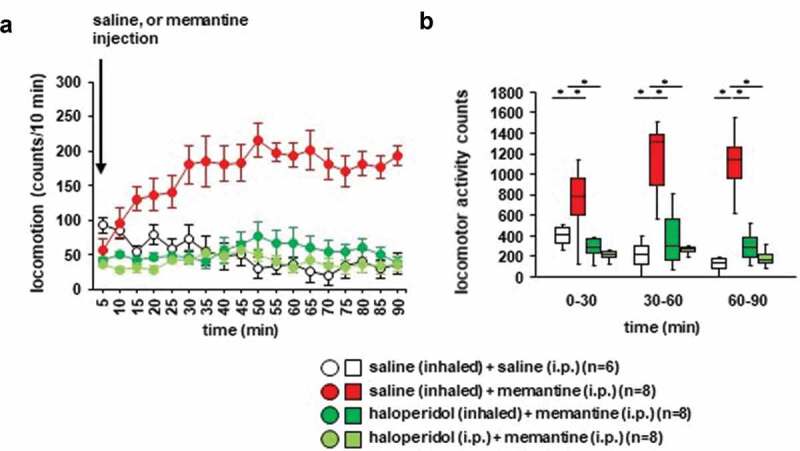
Effect of haloperidol inhalation on memantine-induced locomotion Mice were pretreated with inhaled saline or haloperidol or i.p. with haloperidol 30 minutes before the memantine injection. a) After i.p. memantine administration, the spontaneous locomotor activity of the mice was measured for each 5-minute period for 90 minutes in the locomotor activity test. b) The graphs show the total beam breaks for each 30-minute period after administration.

Whether haloperidol inhalation affected memantine-induced locomotor activity was examined. After the memantine injection, locomotor activity gradually increased (Figure 4(a): *F*_17,476_ = 5.212, *p* < 0.001; saline vs. memantine, *p* < 0.001; saline vs. haloperidol (inhaled), *p* = 0.992; saline vs. haloperidol (i.p.), *p* = 0.930; memantine vs. haloperidol (inhaled), *p* < 0.001; memantine vs. haloperidol (i.p.), *p* < 0.001; haloperidol (inhaled) vs. haloperidol (i.p.), *p* = 0.813). In contrast, locomotor activity gradually decreased after the injection of saline. Haloperidol pretreatment (i.e., injection of haloperidol or inhalation of haloperidol) had an effect on basal locomotor activity, and haloperidol prevented the increase of locomotor activity induced by memantine (Figure 4(a,b)), for 0–30 minutes: *F*_3,31_ = 11.403, *p* < 0.001; saline vs. memantine, *p* = 0.027; saline vs. haloperidol (inhaled), *p* = 0.363; saline vs. haloperidol (i.p.), *p* = 0.106; memantine vs. haloperidol (inhaled), *p* < 0.001; memantine vs. haloperidol (i.p.), *p* < 0.001; haloperidol (inhaled) vs. haloperidol (i.p.), *p* = 0.891; for 30–60 minutes: *F*_3,31_ = 22.929, *p* < 0.001; saline vs. memantine, *p* < 0.001; saline vs. haloperidol (inhaled), *p* = 0.789; saline vs. haloperidol (i.p.), *p* = 0.997; memantine vs. haloperidol (inhaled), *p* < 0.001; memantine vs. haloperidol (i.p.), *p* < 0.001; haloperidol (inhaled) vs. haloperidol (i.p.), *p* = 0.886; for 60–90 minutes: *F*_3,31_ = 28.325, *p* < 0.001; saline vs. memantine, *p* < 0.001; saline vs. haloperidol (inhaled), *p* = 0.671; saline vs. haloperidol (i.p.), *p* = 0.996; memantine vs. haloperidol (inhaled), *p* < 0.001; memantine vs. haloperidol (i.p.), *p* < 0.001; haloperidol (inhaled) vs. haloperidol (i.p.), *p* = 0.802). No significant difference was observed in the locomotor activity counts between mice exposed to haloperidol injection and mice exposed to haloperidol inhalation (Figure 4(a,b)). Haloperidol administered 30 minutes before the i.p. injection of memantine did not increase locomotor activity.

* Indicates a significant difference between groups (*p* < 0.05). The *p* values were calculated using **A** two-way repeated measures analysis of variance and **B** two-way analysis of variance.(**A–B**) control: n = 6, memantine: n = 8, haloperidol (inhaled): n = 8, haloperidol (i.p.): n = 8.

### 3.4. Effects of betahistine inhalation on MK-801–induced locomotor activity in mice

**Figure 5.**
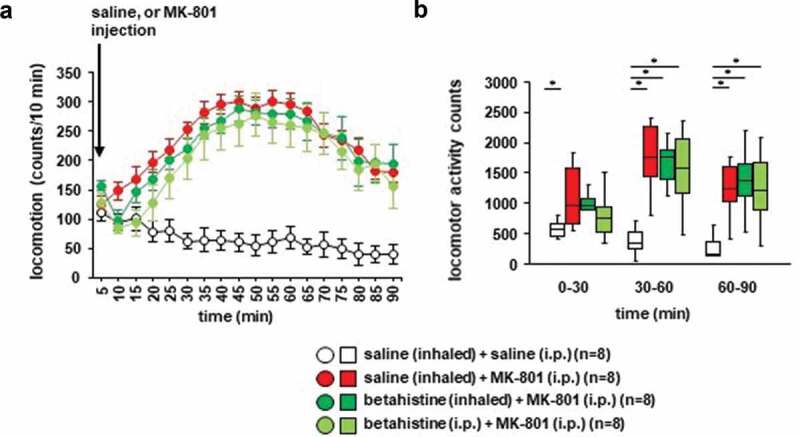
The effect of betahistine inhalation on MK-801-induced locomotion Mice were pretreated with inhaled saline or betahistine or i.p. with betahistine 30 minutes before the MK-801 injection. a) After i.p. MK-801 administration, the spontaneous locomotor activity of the mice was measured for each 5-minute period for 90 minutes in the locomotor activity test. b) The graphs show the total beam breaks for each 30-minute period after administration.

We examined whether betahistine inhalation affected MK-801-induced locomotor activity. Locomotor activity gradually increased after the MK-801 injection (Figure 5(a)): *F*_17,442_ = 5.134, *p* < 0.001; saline vs. MK-801, *p* = 0.001; saline vs. betahistine (inhaled), *p* = 0.003; saline vs. betahistine (i.p.), *p* = 0.011; MK-801 vs. betahistine (inhaled), *p* = 0.985; MK-801 vs. betahistine (i.p.), *p* = 0.816; betahistine (inhaled) vs. betahistine (i.p.), *p* = 0.952). In contrast, locomotor activity gradually decreased after the injection of saline. Betahistine pretreatment (i.e., injection of betahistine or inhalation of betahistine) did not change MK-801-induced locomotor activity (Figure 5(a,b), for 0–30 minutes: *F*_3,31_ = 3.175, *p* = 0.041; saline vs. MK-801, *p* = 0.034; saline vs. betahistine (inhaled), *p* = 0.122; saline vs. betahistine (i.p.), *p* = 0.503; MK-801 vs. betahistine (inhaled), *p* = 0.913; MK-801 vs. betahistine (i.p.), *p* = 0.383; betahistine (inhaled) vs. betahistine (i.p.), *p* = 0.768; for 30–60 minutes: *F*_3,31_ = 11.123, *p* < 0.001; saline vs. MK-801, *p* < 0.001; saline vs. betahistine (inhaled), *p* < 0.001; saline vs. betahistine (i.p.), *p* = 0.001; MK-801 vs. betahistine (inhaled), *p* = 0.967; MK-801 vs. betahistine (i.p.), *p* = 0.853; betahistine (inhaled) vs. betahistine (i.p.), *p* = 0.986; for 60–90 minutes: *F*_3,31_ = 5.913, *p* = 0.003; saline vs. MK-801, *p* = 0.006; saline vs. betahistine (inhaled), *p* = 0.006; saline vs. betahistine (i.p.), *p* = 0.012; MK-801 vs. betahistine (inhaled), *p* = 1.0; MK-801 vs. betahistine (i.p.), *p* = 0.989; betahistine (inhaled) vs. betahistine (i.p.), *p* = 0.989). No significant difference was observed in the locomotor activity counts between mice exposed to betahistine and mice not exposed to betahistine (Figure 5(a,b)). Betahistine administered 30 minutes before the i.p. injection of MK-801 did not change MK-801-induced locomotor activity.

* Indicates a significant difference between groups (*p* < 0.05). The *p* values were calculated **A** using two-way repeated measures analysis of variance and **B** two-way analysis of variance.(**A–B**) control: n = 8, MK-801: n = 8, betahistine (inhaled): n = 8, betahistine (i.p.): n = 8.

### 3.5. Effects of betahistine inhalation on memantine-induced locomotor activity in mice

**Figure 6.**
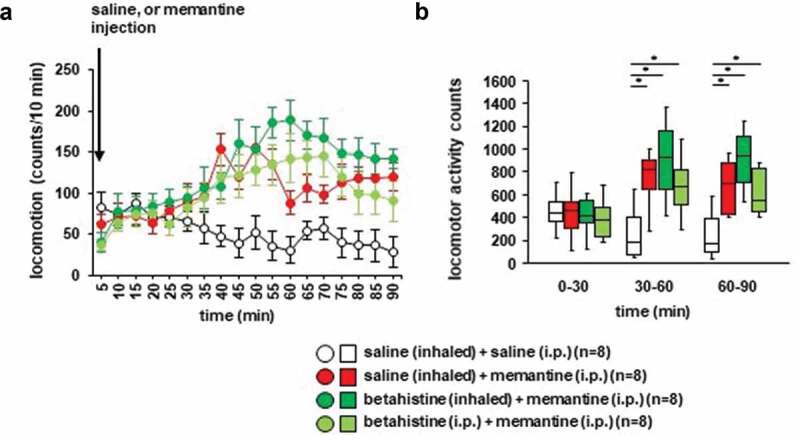
Effect of betahistine inhalation on memantine-induced locomotion Mice were pretreated with inhaled saline or betahistine or i.p. with betahistine 30 minutes before the memantine injection. a) After i.p. memantine administration, spontaneous locomotor activity in the mice was measured for each 5-minute period for 90 minutes in the locomotor activity test. b) The graphs show the total beam breaks for each 30-minute period after administration.

We examined whether betahistine inhalation affected memantine-induced locomotor activity. Locomotor activity gradually increased after the memantine injection (Figure 6(a): *F*_17,442_ = 3.864, *p* < 0.001; saline vs. memantine, *p* = 0.095; saline vs. betahistine (inhaled), *p* = 0.007; saline vs. betahistine (i.p.), *p* = 0.112; memantine vs. betahistine (inhaled), *p* = 0.594; memantine vs. betahistine (i.p.), *p* = 1.0; betahistine (inhaled) vs. betahistine (i.p.), *p* = 0.537; Figure 6(b), for 0–30 minutes: *F*_3,31_ = 0.187, *p* = 0.904; saline vs. memantine, *p* = 0.999; saline vs. betahistine (inhaled), *p* = 1.0; saline vs. betahistine (i.p.), *p* = 0.945; memantine vs. betahistine (inhaled), *p* = 0.995; memantine vs. betahistine (i.p.), *p* = 0.968; betahistine (inhaled) vs. betahistine (i.p.), *p* = 0.895; for 30–60 minutes: *F*_3,31_ = 5.488, *p* = 0.005; saline vs. memantine, *p* = 0.031; saline vs. betahistine (inhaled), *p* = 0.003; saline vs. betahistine (i.p.), *p* = 0.033; memantine vs. betahistine (inhaled), *p* = 0.726; memantine vs. betahistine (i.p.), *p* = 1.0; betahistine (inhaled) vs. betahistine (i.p.), *p* = 0.707; for 60–90 minutes: *F*_3,31_ = 6.539, *p* = 0.002; saline vs. memantine, *p* = 0.046; saline vs. betahistine (inhaled), *p* = 0.001; saline vs. betahistine (i.p.), *p* = 0.034; memantine vs. betahistine (inhaled), *p* = 0.323; memantine vs. betahistine (i.p.), *p* = 0.999; betahistine (inhaled) vs. betahistine (i.p.), *p* = 0.396). In contrast, locomotor activity gradually decreased after the injection of saline. Betahistine pretreatment (i.e., injection of betahistine or inhalation of betahistine) did not change memantine-induced locomotor activity (Figure 6(a,b)). No significant difference was observed in the locomotor activity counts between mice exposed to betahistine and mice not exposed to betahistine (Figure 6(a,b)). Administration of betahistine 30 minutes before the i.p. injection of memantine did not change memantine-induced locomotor activity.

* Indicates a significant difference between groups (*p* < 0.05). The *p* values were calculated using **A** two-way repeated measures analysis of variance and **B** two-way analysis of variance.(**A–B**) control: n = 8, memantine: n = 8, betahistine (inhaled): n = 8, betahistine (i.p.): n = 8.

## 4. Discussion

To the best of our knowledge, this is the first study to demonstrate that the inhalation of haloperidol suppresses MK-801-induced hyperactivity in mice. Inhalation of haloperidol suppressed the MK-801-induced or memantine-induced increase in locomotor activity. This study provides new insights for the development of novel administration methods of haloperidol.

The present findings are consistent with those of a previous report showing that the i.p. administration of MK-801 or memantine increased the locomotor activity of mice. As with all other pharmacological agents that induce locomotor sensitization, MK-801 has been associated with enhancing dopamine release in the nucleus accumbens [48]. Memantine is a moderate-affinity, uncompetitive NMDA receptor antagonist, while MK-801 is a non-competitive NMDA receptor antagonist [49]. It is possible that the locomotor activity of the mice was increased through the blocking of NMDA receptors by these agents [49].

Haloperidol is a dopamine blocker. It has long been known that the i.p. administration of haloperidol attenuates the MK-801-induced increase in the locomotor activity of mice [50–53]. Haloperidol can block the motor stimulation induced by MK-801 in the mouse brain [54]. We showed that the inhalation of haloperidol inhibited the increased locomotor activity of mice induced by i.p. administration of MK-801. The results of this study indicated that inhaled haloperidol acts on the CNS and takes approximately 30 minutes to exert its effect in the mouse brain.

Memantine activates the dopamine D2 receptor [34] and increases the amount of locomotor activity in mice [35]. Some research indicates that the increase in mouse locomotor activity by the i.p. administration of memantine is suppressed by haloperidol in a dose-dependent manner [35]. Our findings revealed that the increase in locomotor activity of mice induced by the i.p. administration of memantine was suppressed by pretreatment with haloperidol inhalation. The results of this study also showed that, approximately 30 minutes after inhalation, haloperidol reached the brain and exerted its effect. In previous studies [55], nasally administered haloperidol was rapidly absorbed and reached concentrations associated with pharmacological effects and clinically relevant therapeutic effects within 30 minutes. Furthermore, one report [56] demonstrated no detectable haloperidol in the plasma of rats following the intranasal administration of haloperidol. That report suggested that the drug is transported directly from the intranasal cavity to the brain and not via absorption into the systemic circulation. The present study showed that haloperidol acts on the CNS following inhalation, as well as following intranasal and i.p. administration. Further studies are needed to investigate the possible side effects of haloperidol inhalation.

Increased brain histamine levels in mice with memantine-induced hyperactivity has been reported [42]. One report [35] showed that betahistine reduces the increased locomotor activity induced by memantine in mice. However, in the current study, betahistine administered i.p. or by inhalation did not suppress the increase in MK-801- or memantine-induced locomotor activity. This finding is different from that of previous reports. The difference between these studies may be related to the differences in subjects’ body weight, the mouse strains used, and the manufacturers of the administered drugs. Other experiments are needed to clarify the effects of these differences.

Further studies are also needed to clarify the details of the access pathway and the amount of haloperidol that reaches the brain. Major pathways by which it might reach the brain are (1) from the nasal cavity to the brain through the olfactory nerve pathway and the trigeminal nerve pathway; (2) from the nasal cavity and absorption into the bloodstream, thereby penetrating the BBB; and (3) systemic administration and absorption into the bloodstream. Some investigators have suggested that the intranasal route can deliver drugs directly from the nasal cavity along the olfactory and trigeminal nerves to the brain (Tyler et al. 2018). Interestingly, studies that have directly compared brain access from intranasal and intravenous administration routes have interestingly indicated, through brain imaging, efficient transport patterns to specific deep brain regions and interstitial fluid after intranasal administration [57]. Furthermore, investigators have reported that drug molecules can be absorbed systemically from the nasal cavity and penetrate the BBB if the drug molecules have sufficient lipophilicity [58]. In fact, alternative routes of transmucosal delivery have been used in other areas of medicine and drug therapy when rapid absorption and action are desired. Buccal and intranasal delivery products have been used to treat pain, migraine headache, spasticity, and angina [10, 11]. The application of transmucosal routes (e.g., intranasal delivery) of medications for psychiatric emergencies, such as the treatment of acute excitement and psychosis, may enhance the rapid absorption and subsequent effects of antipsychotics. The results of this study demonstrated that the administration of haloperidol by inhalation is an attractive option for delivering drugs to the brain, and that delivery by inhalation is a better option for targeting drugs to the brain.

Further studies are needed to control the amount of inhaled haloperidol that reaches the brain. However, for inhaled anesthetics and bronchoactive drugs, the drug concentration in the blood differs distinctly, depending on the intake concentration and the inhalation time [59, 60]. The inhalation of haloperidol may be similarly controlled.

## 5. Conclusions

In this study, we showed that the inhalation of a drug solution containing haloperidol could act on the CNS of mice. The results of this study provide new insights into the development of new dosing regimens for haloperidol.

## Data Availability

All relevant data are within the manuscript.
